# Genetic variability and associations of Trakehner and other horse populations in Lithuania

**DOI:** 10.5194/aab-69-323-2026

**Published:** 2026-06-02

**Authors:** Alma Račkauskaitė, Rūta Šveistienė, Violeta Razmaitė, Virginija Jatkauskienė, Šarūnė Marašinskienė

**Affiliations:** 1 Department of Animal Breeding and Reproduction, Animal Science Institute, Lithuanian University of Health Sciences, Baisogala, 82317, Lithuania

## Abstract

To obtain genetic parameters relevant for the management of animal genetic resources, this study provides a comparative characterization of the Trakehner (TRAK) horse population in relation to four other horse populations maintained in Lithuania – Arabian (ARAB), Baltic Warmblood (BW), Lithuanian Warmblood (LW), and Zemaitukai (ZEM). These populations represent diverse histories, selection goals, and breeding practices. Pedigree data ( n= 22 666) and parentage verification genetic data based on blood group markers ( n= 1293) were used to evaluate genetic diversity, population structure, and inter-population relationships. Despite a 25 % population decline in recent years, the TRAK population maintained moderate inbreeding ( F= 0.037) and a balanced genetic structure. The effective population size ( $N_{e}$ ) was lower than in BW and LW but higher than in the endangered ZEM population. TRAK retained a high number of alleles ( $N_{a}=$ 3.83), with observed heterozygosity ( $H_{o}=$ 0.460) slightly lower than that observed in open sport horse populations. Comparison of all individuals and the Lithuanian reference populations revealed stable or increased heterozygosity in currently breeding individuals, particularly within TRAK. Genetic distances and assignment tests confirmed close relationships among TRAK, BW, and LW, reflecting shared ancestry. In contrast, ARAB and ZEM populations remained genetically distinct. PCA and heatmap analyses further supported the separation between closed and open populations in the current breeding context. These findings highlight that population structure, historical gene flow, and management practices jointly shape genetic variability. The results can be used to optimize mating plans by balancing sire and dam contributions, monitor inbreeding and effective population size trends, manage gene flow between related populations (TRAK, BW, LW), and develop targeted conservation strategies for genetically distinct breeds such as ZEM and ARAB.

## 1 Introduction

Sport horse breeding is focused on the improvement of the genetic ability of warmblood riding horses for performance in competitions. In open subpopulations, an extensive exchange of the breeding stock between breeding organizations has been going on for decades. Significant improvements in reproduction techniques (e.g. chilled and frozen semen) have further facilitated the simultaneous use of breeding stallions in multiple countries and a large exchange of genetic material as a consequence (Koenen et al., 2004). The liberalization of European animal breeding legislation and an increasing diversity of equestrian sports have led to a constant rise in the number of horse breeds and breed registries. In addition, there is a trend towards an international expansion of the bigger established sport horse breeds (Aurich and Aurich, 2006). In this way, the importation of foreign genetic material (from foreign sport horse breeds) is present in open populations and gives the breeders the opportunity to choose foreign stallions that best contribute to the improvement of their horses' performance, as regards their physical, physiological, and/or genealogical characteristics (Bartolomé et al., 2011).

Still, there are some sufficiently closed studbooks which aim to preserve genetic resources (Zechner et al., 2002; Pjontek et al., 2012; Cosenza et al., 2019). In closed populations, genetic progress is slower, and it is difficult to obtain high-performance offspring while maintaining the genetic structure and diversity within the breed. Considering sport horse breeds, the Trakehner (TRAK) studbook represents a predominantly closed breeding system based on purebreeding (Trakehner Verband, 2025), whereas most contemporary sport horse breeding programmes operate as open studbooks, facilitating continuous genetic exchange across populations (Doyle et al., 2022). The TRAK breed is one of the oldest riding horse breeds and has long been acknowledged as an original breed, even internationally. Genetically, the breed can be traced back directly to the foundation of the Main Stud in Trakehnen (East Prussia) by a royal decree of the Prussian King in 1732. TRAK are a riding horse breed bred in accordance with the principles of pure breeding, with a high genetic proportion of English and Arab thoroughbred, Shagya, and Anglo-Arab, in keeping with the selection criteria established for the breed (Trakehner Verband, 2022). In other sport horse breeding programmes, TRAK stallions are commonly used for crossbreeding mares (Schröder et al., 2010; Roos et al., 2015; Oldenburger Pferdezuchtverband, 2022). In Lithuania TRAK were used for cavalry and sport (Krikščiūnas and Skaisgiris, 1939). Nowadays, according to the data of the Lithuanian Farm Animal Register, the total population of TRAK in Lithuania decreased by 25 % from 2014 to 2021.

Historical records indicate that the foundation of the Trakehnen Stud involved horses from studs located in Lithuania Minor (East Prussia), such as the Sereitlaukis estate (Račkauskaitė et al., 2021). Therefore, it could be assumed that local Lithuanian horses, including the Zemaitukai (ZEM), could have contributed to the early formation of the TRAK breed. ZEM horses have been known since the 6th–7th centuries (Gleß, 1989). Until the middle of the 19th century, the breed was dominant in the country. However, more intensive agricultural development has led to crossbreeding and dangerous declines of the population. After World War II in the first Lithuanian state studbook (1948), barely 17 purebred ZEM were entered; in 1994, only 30 horses were left (Macijauskienė et al., 2009). To restore the population, the studbook was opened, and three stallions of historically related breeds (Lithuanian large type Zemaitukai, Estonian native, and Arab) were used for a very low number of mares (Macijauskienė, 2009). The population of ZEM horses has increased, but the breed is still classified as endangered.

The Arabian (ARAB) horse is another breed of closed populations and one of the world's oldest breeds of any domesticated animals (Cosgrove et al., 2020), but the history of the breed's modern populations, described in pedigree records, is no longer than 200 years (Głażewska, 2010). The first European studs in Poland, Germany, and Hungary were established in the early nineteenth century. In Lithuania, ARAB were already used for breeding in 1937 (Krikščiūnas and Skaisgiris, 1939). Lithuania became a member of the World Arab Horse Organization (WAHO) in 1994. In 2007, 65 ARAB horses were already kept in the breeding nucleus of the national stud (Barisevičius et al., 2007). The breed is listed as transboundary (FAO, 2022).

In 1999, the development of a promising horse population suitable for all classical equestrian disciplines was initiated in Lithuania. This population was maintained as an open breeding structure, allowing local sport horse mares and stallions from various foreign breeds to be included. By 2007, six genetically dominant breed groups were identified within the population based on genotype composition: Hanoverian, TRAK, Holsteiner, Thoroughbred, Budyonny, ARAB, and others (Šveistienė, 2007). According to the World Breeding Federation for Sport Horses (WBFSH), 10 domestic and 30 foreign-approved stallions were used in the breeding programme of the Lithuanian Riding Horse in 2020 (WBFSH, 2021). In 2024, the breed was officially renamed to Lithuanian Warmblood horse (LW) to reflect its composition and alignment with international sport horse classification.

In 2000, the development of the second riding horse population was launched, and the studbook of the Baltic Hanoverian horse was established. The foundation of the Baltic Hanoverian population was a cross between Hanoverian and other sport horses. Hanoverians, TRAK, and Thoroughbreds were the main breeds of light-riding horses between World Wars I and II in Lithuania (Krikščiūnas and Skaisgiris, 1939). The national stud in Zagare became the basis for Hanoverian horse breeding for several decades. In 2022, the name of the studbook was changed to Baltic Warmblood (BW), and currently many other international breeds are used. The studbook registry of the breed is administered in accordance with the rules of WBFSH.

Characterization of animal genetic resources is essential for informed decision-making in conservation and breeding programmes (FAO, 2007; Kierkegaard et al., 2020). In horse populations, pedigree data remain a valuable tool for assessing genetic structure and variability, particularly when molecular information is limited or unavailable. Pedigree-based approaches have been widely applied to analyse genetic diversity, track founder contributions, and assess inbreeding trends, thereby providing insight into the effects of selection, breed formation, and population openness (Duru, 2017; Bokor et al., 2022; Próchniak et al., 2024). In Lithuania, pedigree analyses were complemented by immunogenetic tools, including blood group and protein polymorphism typing, which were introduced in 1985 and applied in breeding control and the characterization of local breeds (Krikščiūnas, 1990). Although these methods are no longer routinely used in many countries, the standardized and long-term dataset they produced allows for the evaluation of genetic relationships and intra- and inter-population diversity. Moreover, these data may serve as a valuable reference for future molecular studies, especially in native and regional horse populations where genomic data remain limited.

Genetic variability under tightly controlled breeding in a declining population is essential. This study aimed to characterize the genetic variability of the TRAK population and its relationships with other horse populations in Lithuania by applying pedigree and parentage verification data. The results provide important insights into the effects of breed formation history and breeding practices on genetic structure, supporting informed decisions in the conservation and sustainable use of equine genetic resources.

## 2 Material and methods

### 2.1 Pedigree data analysis

The pedigree dataset included 22 666 horses from the Lithuanian populations of ARAB, BW, LW, TRAK, and ZEM, with records provided by the Agricultural Data Center. Table 1 presents an overview of the populations analysed. The reference population comprised horses registered in the respective studbooks and of breeding age (3–20 years). Horses outside this age range, as well as geldings, were excluded from the reference population.

**Table 1:** Overview of studbook-based horse populations included in the pedigree analysis.

Studbook registry	Reference population		Entire pedigree file	
	Stallions	Mares	Birth dates	Total individuals
TRAK	312	535	1923–2022	4290
ARAB	44	48	1948–2020	686
BW	500	721	1937–2021	8888
LW	544	738	1936–2021	7450
ZEM	353	481	1938–2021	1352

TRAK, Trakehner; ARAB, Arabian; BW, Baltic Warmblood; LW, Lithuanian Warmblood; and ZEM, Zemaitukai.

The number of founders in each horse population was estimated by summing individuals with unknown parentage, those with only maternal information, and those with only paternal information. The calculation considered the completeness of genealogical records and was based solely on pedigree data from the respective studbooks. This constraint limited the precision of founder estimates. Newly imported horses registered in the studbooks typically have pedigree information extending back only four generations, unless their ancestors were already recorded in the national studbooks.

Subsequent pedigree analysis parameters were calculated using the POPREP software system (Groeneveld et al., 2009).

Pedigree completeness quantifies the proportion of known ancestors across generations and provides a statistical measure of data quality. This parameter is essential, as estimates of inbreeding and effective population size depend heavily on the depth and completeness of pedigree information. In this study, pedigree completeness was calculated using the following formula:

1 $$I_{d_{k}}=\frac{1}{d}\sum_{i=1}^{d}a_{i},$$

where d is the number of generations considered, k represents the paternal or maternal line of the individual I , and $a_{i}$ is the proportion of known ancestors in the generation i (MacCluer et al., 1983).

The number of breeding males and females was determined for a given time period, as this directly influences the genetic structure of the subsequent generation.

The rate of inbreeding per generation ( ΔF ) was calculated using

2 $$\Delta F=\frac{F_{t}-F_{t-1}}{1-F_{t-1}},$$

where $F_{t}$ and $F_{t-1}$ are the average inbreeding of offspring and their parents, respectively (Falconer and Mackay, 1996).

Several methods have been proposed to compute the effective population size ( $N_{e}$ ). $N_{e}$ via reproductive animals (Falconer and Mackay, 1996) was estimated based on the numbers of breeding animals using

3 $$N_{e}=\frac{4N_{m}N_{f}}{N_{m}+N_{f}}\cdot 0.7,$$

where $N_{m}$ and $N_{f}$ are the number of male and female parents, respectively.

$N_{e}$ via ΔF was calculated using

4 $$N_{e}=\frac{1}{2}\Delta F.$$

To assess breeding intensity and genetic progress, the generation interval was calculated. It was defined as the average age of parents at the birth of their offspring that were retained for breeding. Generation intervals were estimated for four parental pathways: sire to sire, sire to dam, dam to sire, and dam to dam. The overall generation interval was used for comparison among horse populations.

### 2.2 Genetic data analysis

#### 2.2.1 Dataset

Genetic data from routine parentage verification tests, based on blood typing, were used in this study. A total of 1293 horses were included in the analysis. The analysis was conducted for two datasets: all genetically tested horses, to assess the overall genetic diversity, and the reference populations, consisting of living animals still eligible for breeding and contributing to the next generation (Table 2).

**Table 2:** Composition of horse populations analysed by genetic parentage verification.

Horse	Birth dates	Total number	Number of
population		of analysed	analysed horses
		horses	from reference
			population
TRAK	1984–2018	302	81
ARAB	1987–2017	208	30
BW	1982–2017	98	12
LW	1980–2009	155	22
ZEM	1975–2020	476	156

TRAK, Trakehner; ARAB, Arabian; BW, Baltic Warmblood; LW, Lithuanian Warmblood; and ZEM, Zemaitukai.

Horses were individually selected and grouped; for each animal, pedigree information (up to four generations) was reviewed to assess breed composition of the tested individuals and their parents. Efforts were made to select individuals as unrelated as possible, considering both maternal and paternal lineage. However, due to the small size of some subpopulations, it was not always possible to avoid close relationships. In a few cases, the same horse appeared in more than one population due to its use across multiple breeding programmes.

Blood typing was carried out at the Laboratory for Genetic Studies of the Animal Science Institute of Lithuanian University of Health Sciences. The samples for analysis were delivered by various horse breeders of Lithuania. Agglutination and haemolysis reactions were carried out by the requirements of the International Society for Animal Genetics (ISAG). Blood groups were identified using seven genetic systems: EAA, EAC, EAD, EAK, EAP, EAQ, and EAU. The following alleles were tested: $A^{a}$ , $A^{abd}$ , $A^{ad}$ , $A^{b}$ , $A^{bc}$ , $A^{c},C^{a},D^{ad}$ , $D^{adl}$ , $D^{bcm}$ , $D^{cfgm}$ , $D^{cfm}$ , $D^{cgm}$ , $D^{dfk}$ , $D^{dghm}$ , $D^{dk}$ , $D^{dkl}$ , $D^{dl}$ , $K^{a}$ , $P^{b}$ , $Q^{a}$ , $Q^{abc}$ , $Q^{ac}$ , $Q^{b}$ , $Q^{c}$ , and $U^{a}$ . Five blood protein genetic systems were identified: albumin (*Al*), vitamin-D-binding protein (*Gc*), serum esterase (*Es*), postalbumin (*Xk*), and transferrin (*Tf*). The following alleles were analysed: *Al* system (*A*, *B*), *Gc* (*F, S*), *Es* (*F, I, S, X*),* Xk* (*F, K, S*), and *Tf* (*D, F, H, O, R*) (Juras et al., 2003).

#### 2.2.2 Genetic diversity parameters

Genetic diversity measures were calculated using GENALEX v6.501 (Peakall and Smouse, 2012). For each locus, the number of alleles ( $N_{a})$ , the effective number of alleles ( $A_{e}$ ), and observed ( $H_{o}$ ) and expected heterozygosity ( $H_{e}$ ) were determined. Deviations from the Hardy–Weinberg equilibrium (HWE) were tested using χ -squared test P values.

Across horse populations, allele frequencies and average values of genetic diversity parameters were computed, including mean $N_{a}$ , $A_{e}$ , $H_{o}$ , and $H_{e}$ , across all loci. Additionally, tests for deviations from HWE were performed for each population. An analysis of molecular variance (AMOVA) among horse populations was also conducted to evaluate genetic differentiation.

#### 2.2.3 Relationships of horse populations

Genetic relationships among horse populations were evaluated using multiple complementary approaches. The pairwise genetic differences among horse populations were evaluated based on Nei's standard genetic distance ( D ), and pairwise values of the fixation index ( $F_{\mathrm{st}}$ ) were determined using FSTAT v2.9.3 (Goudet, 2001).

To explore the assignment of individuals to populations, a likelihood-based population assignment test was conducted in GENALEX following the method of Paetkau et al. (2004). This test uses allele frequencies across loci to estimate the probability of an individual belonging to a given population, thereby identifying the population with the highest likelihood of origin. Such methods can also provide insights into gene flow between subpopulations (Remais et al., 2011). Results of the assignment test were visualized using the Circos software (Krzywinski et al., 2009), which displays connections via circular ideograms.

To further examine population structure, principal component analysis (PCA) was performed using a symmetric correlation matrix of allele frequencies. The PCA plot was generated using Minitab version 15 (Minitab, LLC, State College, PA, USA).

Additionally, a heatmap with hierarchical clustering was generated to visualize genetic similarities and changes over time. Both alleles (rows) and horse populations (columns) were clustered using Euclidean distance and the average linkage method. The analysis included both reference populations (currently breeding animals) and full population datasets (historical gene pool) to assess temporal shifts in genetic composition. The heatmap was produced using ClustVis (Metsalu and Vilo, 2015).

## 3 Results

### 3.1 Population structure by genealogical data

In 2020, pedigree completeness six generations deep was the highest in the closed ZEM population (96.9 %), followed by TRAK (82.0 %) and ARAB (72.4 %) (Table 3). In contrast, open population BW and LW exhibited lower completeness (39.9 % and 54.5 %, respectively), reflecting the frequent use of imported animals with limited pedigree depth. The number of founders was the smallest in ARAB (8) and ZEM (23), while BW and LW had markedly more (431 and 592), consistent with their open breeding structure.

**Table 3:** Pedigree completeness and founders in analysed horse populations.

Population	Pedigree completeness	Founders	Founders	Founders
	for six generations	with unknown	with only females	with only males
	deep, %	parents	known parents	known parents
TRAK	82.0	85	13	20
ARAB	72.4	2	1	5
BW	39.9	320	67	44
LW	54.5	471	56	65
ZEM	96.9	18	3	2

TRAK, Trakehner; ARAB, Arabian; BW, Baltic Warmblood; LW, Lithuanian Warmblood; and ZEM, Zemaitukai.

Table 4 summarizes the parameters characterizing breeding intensity and population structure. Despite a declining trend, the TRAK population had 78 reproductive mares and 21 stallions, resulting in an effective population size ( $N_{e}$ ) of 211. Compared to open populations BW and LW, which showed higher $N_{e}$ values via breeding animals (851 and 758, respectively), TRAK had a more balanced and closed structure. However, BW and LW also exhibited a large drop in $N_{e}$ when calculated via inbreeding rate (311 and 216), reflecting hidden relatedness and unequal parental contributions.

**Table 4:** Breeding animal usage, genetic diversity indicators, and generation intervals characterizing the structure and sustainability of horse populations.

Population	Sires used	Dames used	$N_{e}$ via	F	ΔF	$N_{e}$ via	Generation
	to get offspring	to get offspring	breeding horses			ΔF	interval
TRAK	21	78	211	0.0373	0.0033	153	12
ARAB	3	4	52	–	–	43	10
BW	56	113	851	0.0008	0.0016	311	11
LW	45	106	758	0.0089	0.0023	216	13
ZEM	41	74	223	0.2342	0.0265	19	10

TRAK, Trakehner; ARAB, Arabian; BW, Baltic Warmblood; LW, Lithuanian Warmblood; ZEM, Zemaitukai; F , rate of inbreeding; ΔF , rate of inbreeding per generation; and $N_{e}$ , effective population size.

In contrast, the endangered ZEM breed showed a dramatic discrepancy between $N_{e}$ via breeding animals (223) and via inbreeding rate (19), reflecting high inbreeding pressure ( F= 0.234; ΔF= 0.0265). The TRAK population demonstrated moderate inbreeding ( F= 0.0373; ΔF= 0.0033), indicating controlled mating strategies.

The mean generation interval in TRAK was 12 years – comparable to LW (13) and BW (11) – but 2 years longer than in ARAB and ZEM populations (10 years each). A longer generation interval, typical in sport horse breeding, contributes to slower genetic turnover but may help stabilize diversity in closed populations like TRAK.

### 3.2 Genetic diversity

#### 3.2.1 Total genetic diversity of horse blood systems

In total, 49 alleles were detected at 12 blood group loci across all studied horses (Table 5). The most polymorphic locus was *EAD*, with 12 alleles and an $A_{e}$ value of 7.364 and the highest $H_{e}$ value of 0.864. Across all horses, the average $H_{o}$ was 0.475 and $H_{e}$ was 0.395, indicating a moderate level of diversity. The χ -squared test for Hardy–Weinberg equilibrium (HWE) showed statistically significant results on eight loci.

**Table 5:** Summary statistics of diversity indices per blood group locus in all/reference horses.

Locus	$N_{a}$	$A_{e}$	$H_{o}$	$H_{e}$	Prob	Signif.
*EAA*	7 / 7	2.966 / 2.931	0.938 / 0.950	0.663 / 0.659	0.000 / 0.000	${}^{***}{/}^{***}$
*EAC*	2 / 2	1.979 / 1.985	0.897 / 0.914	0.495 / 0.496	0.000 / 0.000	${}^{***}{/}^{***}$
*EAD*	12 / 12	7.364 / 6.633	0.950 / 0.960	0.864 / 0.849	0.000 / 0.000	${}^{***}{/}^{***}$
*EAK*	2 / 2	1.045 / 1.048	0.044 / 0.047	0.043 / 0.045 /	0.433 / 0.680	ns / ns
*EAP*	2 / 2	1.082 / 1.086	0.079 / 0.083	0.076 / 0.080	0.147 / 0.452	ns / ns
*EAQ*	6 / 5	2.466 / 2.582	0.649 / 0.718	0.594 / 0.613	0.000 / 0.000	${}^{***}{/}^{***}$
*EAU*	2 / 2	1.260 / 1.279	0.233 / 0.249	0.206 / 0.218	0.000 / 0.014	${}^{***}{/}^{*}$
*Al*	2 / 2	1.892 / 1.779	0.635 / 0.568	0.471 / 0.438	0.000 / 0.000	${}^{***}{/}^{***}$
*Gc*	2 / 2	1.254 / 1.226	0.194 / 0.164	0.202 / 0.184	0.152 / 0.064	ns / ns
*Es*	4 / 3	1.665 / 1.818	0.363 / 0.435	0.399 / 0.450	0.000 / 0.301	${}^{***}/$ ns
*Xk*	3 / 3	1.049 / 1.065	0.048 / 0.063	0.047 / 0.061	0.864 / 0.956	ns / ns
*Tf*	5 / 5	3.137 / 3.011	0.664 / 0.645	0.681 / 0.668	0.000 / 0.661	${}^{***}/$ ns
Mean	4.083 / 3.917	2.263 / 2.204	0.475 / 0.483	0.395 / 0.397	–	–

TRAK, Trakehner; ARAB, Arabian; BW, Baltic Warmblood; LW, Lithuanian Warmblood; ZEM, Zemaitukai; $N_{a}$ , number of alleles; $A_{e}$ , number of effective alleles; $H_{o}$ , observed heterozygosity; $H_{e}$ , expected heterozygosity; Prob, probability; Signif., significance for HWE; and ns, not significant. ^*^ P < 0.05, ^***^ P ,< 0.001.

Similar results were observed in the reference population of horses eligible for breeding. Despite a smaller sample size, the reference group showed a slightly higher average $H_{o}$ (0.483) and $H_{e}$ (0.397), indicating that genetic diversity is being maintained among actively breeding individuals. Comparable values of $N_{a}$ and $A_{e}$ between the groups support this observation.

#### 3.2.2 Genetic diversity in different horse populations

Allele frequencies and the significance for HWE are presented in Supplement Table S1. The number of alleles identified per population ranged from 44 to 47. The highest allelic diversity was observed in the ZEM population (47 alleles), followed by TRAK and ARAB (both with 46). Although the ARAB population showed a relatively high total allele count, it also exhibited the highest proportion of rare alleles (frequency < 0.1) and the absence of several alleles found in other populations. The BW and LW populations had the lowest numbers of alleles, with 45 and 44, respectively.

Some alleles, such as *EAQb*, were characteristic of Lithuanian riding horse populations and occurred at a very low frequency in ZEM horses. In the TRAK population, the most frequent alleles were *EADdghm* (0.388), *EAQc* (0.357), and *EsF* (0.334). In the ARAB population, the allele *TfO* was the most frequent (0.327).

Table 6 presents the mean values of genetic diversity parameters across all analysed horses and reference populations in the analysed loci. The TRAK population showed a mean number of alleles per locus ( $N_{a}$ ) of 3.833 across all animals and 3.417 in the reference population. The effective number of alleles ( $A_{e}$ ) increased slightly from 2.218 to 2.271 in the reference population, suggesting the maintenance or even slight improvement of allelic richness in the current breeding cohort. However, observed heterozygosity ( $H_{o}$ ) rose from 0.460 to 0.488, while expected heterozygosity ( $H_{e}$ ) increased from 0.375 to 0.392, indicating that recent breeding efforts may be successfully preserving heterozygosity despite closed studbook practices.

**Table 6:** Genetic diversity parameters within five horse populations (all horses/reference horses).

Horse population		$N_{a}$	$A_{e}$	$H_{o}$	$H_{e}$
TRAK	Mean	3.833 / 3.417	2.218 / 2.271	0.460 / 0.488	0.375 / 0.392
	SE	0.806 / 0.690	0.513 / 0.512	0.109 / 0.107	0.083 / 0.080
ARAB	Mean	3.833 / 3.167	1.911 / 184	0.441 / 0.444	0.326 / 0.323
	SE	0.911 / 0.767	0.382 / 0.394	0.117 / 0.115	0.080 / 0.077
BW	Mean	3.750 / 3.083	2.312 / 2.132	0.503 / 0.479	0.414 / 0.373
	SE	0.799 / 0.557	0.498 / 0.410	0.099 / 0.111	0.079 / 0.084
LW	Mean	3.667 / 3.417	2.243 / 2.151	0.498 / 0.489	0.408 / 0.398
	SE	0.732 / 0.668	0.451 / 0.440	0.102 / 0.103	0.077 / 0.073
ZEM	Mean	3.917 / 3.417	2.013 / 1.998	0.485 / 0.487	0.381 / 0.380
	SE	0.811 / 0.753	0.305 / 0.296	0.106 / 0.107	0.079 / 0.078
All populations	Mean	3.800 / 3.300	2.139 / 2.089	0.477 / 0.477	0.381 / 0.373
	SE	0.352 / 0.299	0.190 / 0.181	0.046 / 0.047	0.035 / 0.034

TRAK, Trakehner; ARAB, Arabian; BW, Baltic Warmblood; LW, Lithuanian Warmblood; ZEM, Zemaitukai; $N_{a}$ , number of alleles; $A_{e}$ , number of effective alleles; $H_{o}$ , observed heterozygosity; $H_{e}$ , expected heterozygosity; and SE, standard error.

Open populations BW and LW showed the highest $H_{o}$ and $H_{e}$ in both total and reference populations, reflecting high heterogeneity due to crossbreeding. For instance, BW had $H_{o}$ values of 0.503 (all horses) and 0.479 (reference) and $A_{e}$ values of 2.312 and 2.132, respectively. This slight decrease in the reference population suggests a stabilization trend in the actively used gene pool.

In contrast, the ARAB population exhibited the lowest genetic variability, with $H_{o}$ values of 0.441 in the total population and 0.444 in the reference population, indicating minimal difference and confirming its narrow genetic base. The ZEM breed maintained high $N_{a}$ and $H_{o}$ in both groups, yet a slight decrease in $A_{e}$ (from 2.013 to 1.998) suggests a potential loss of rare alleles in the breeding population.

#### 3.2.3 Relationships of horse populations

Pairwise comparisons of Nei's genetic distance and $F_{\mathrm{st}}$ values, calculated separately for the full dataset and reference populations (Table 7), revealed consistent patterns of genetic relationships. TRAK horses were most closely related to BW and LW, with Nei's genetic distance values of 0.008 and 0.009 (full population) and 0.008 and 0.010 (reference population), respectively. These results reflect historical and ongoing gene flow from TRAK into these open sport horse populations. In contrast, ZEM showed the greatest differentiation from TRAK, with a Nei genetic distance value of 0.045 in both datasets, and was also genetically distinct from all other breeds, consistently exhibiting the highest $F_{\mathrm{st}}$ values across comparisons.

**Table 7:** Pairwise population matrix of Nei's genetic distance below the diagonal and fixation index (Fst) values above the diagonal (all horses/reference horses).

Horse population	TRAK	ARAB	BW	LW	ZEM
TRAK	–	0.019 / 0.024	0.008 / 0.011	0.008 / 0.010	0.028 / 0.029
ARAB	0.015 / 0.021	–	0.023 / 0.019	0.018 / 0.017	0.053 / 0.054
BW	0.009 / 0.009	0.023 / 0.026	–	0.003 / 0.012	0.028 / 0.026
LW	0.006 / 0.006	0.018 / 0.014	0.004 / 0.014	–	0.028 / 0.034
ZEM	0.045 / 0.045	0.082 / 0.081	0.047 / 0.044	0.050 / 0.055	–

TRAK, Trakehner; ARAB, Arabian; BW, Baltic Warmblood; LW, Lithuanian Warmblood; and ZEM, Zemaitukai.

The $F_{\mathrm{st}}$ value between TRAK and ARAB increased from 0.015 in the full dataset to 0.021 in the reference population, while similar increases were observed in other breed pairs. This pattern suggests increasing genetic structuring or divergence within the currently breeding animals, indicating that recent breeding practices may be reinforcing population boundaries or limiting gene exchange.

**Figure 1:**
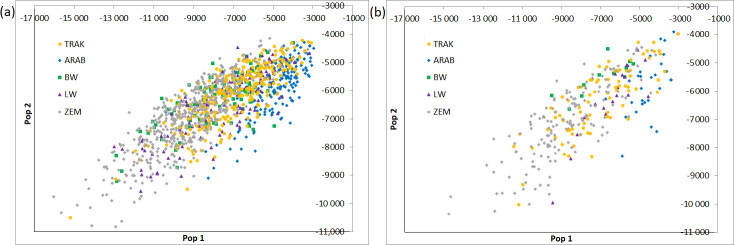
Multidimensional scaling of population assignment probabilities (**a** – all horses, **b** – reference horses).

The population assignment test (Fig. 1) is based on 12 loci. The test calculates the genotype probability of random assignment to each of the populations tested. Two axes are represented in the graph out of the resulting multidimensional plot of probabilities (one axis for each population). The plot gives an overall graphic representation of similarities/dissimilarities among genotypes within and among populations.

ARAB and ZEM populations formed clear, isolated groups in both datasets, confirming strong genetic integrity and minimal gene flow. In contrast, TRAK, BW, and LW horses showed partial overlap, especially in the all-horse dataset, highlighting shared ancestry or historical crossbreeding.

In the reference population, individual assignments became slightly more distinct, suggesting increasing differentiation among breeding groups. TRAK horses shifted toward a more centralized cluster, indicating internal consistency within the actively bred subpopulation.

**Figure 2:**
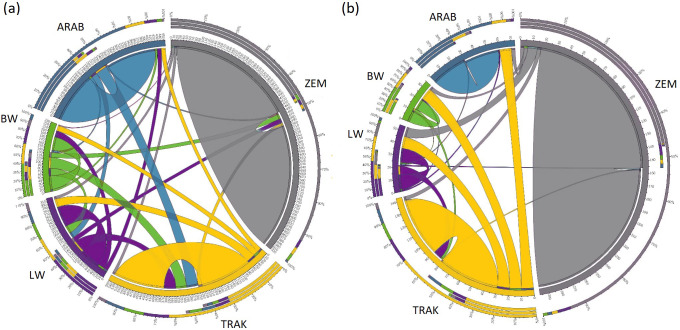
Population purity and admixture proportions revealed by Circos visualization (**a** – all horses, **b** – reference horses).

The Circos plot (Fig. 2) revealed the proportion of individuals assigned to their own or other populations, offering insights into genetic identity and admixture.

In the full dataset, 57.9 % of TRAK horses were correctly assigned to their own population, while 42.1 % were assigned to others – primarily LW (13.6 %) and ARAB (13.2 %). This admixture signal reflects the historical influence of TRAK on sport horse populations and its inclusion of Arab ancestry. In contrast, ZEM and ARAB populations showed strong assignment to their own groups (89 % and 83 %, respectively), indicating low external introgression.

In the reference population, assignment to the breed's own group increased slightly for all breeds. For TRAK, 62.1 % of individuals were assigned to the TRAK group, suggesting that currently used breeding animals are more genetically homogeneous than the historical population.

**Figure 3:**
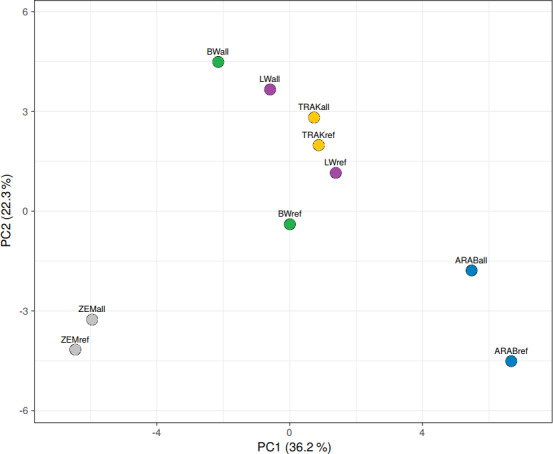
PCA of population-level genetic variation. “ref” – reference population (breeding horses); “all” – all analysed horses (total gene pool).

The PCA of the blood group and protein marker data resulted in two principal components, explaining 36.2 % and 22.3 % of the total variation, respectively (Fig. 3). Four components had eigenvalues > 1, accounting for 100 % of total variation. All correlations between variables and principal components were low, indicating no dominant individual markers.

Visualization of the genetic relationships among populations revealed clear group-specific clustering. TRAK, BW, and LW populations occupied overlapping space, with TRAK more centrally located and compact in the reference dataset. This supports their shared ancestry and highlights gene flow while also suggesting increasing genetic structuring among currently breeding animals.

In contrast, ZEM and ARAB populations formed distinct, non-overlapping clusters, consistent with their closed or isolated breeding histories. The comparison of full and reference datasets confirmed that reference groups are more genetically defined, reflecting recent selection and restricted gene exchange.

**Figure 4:**
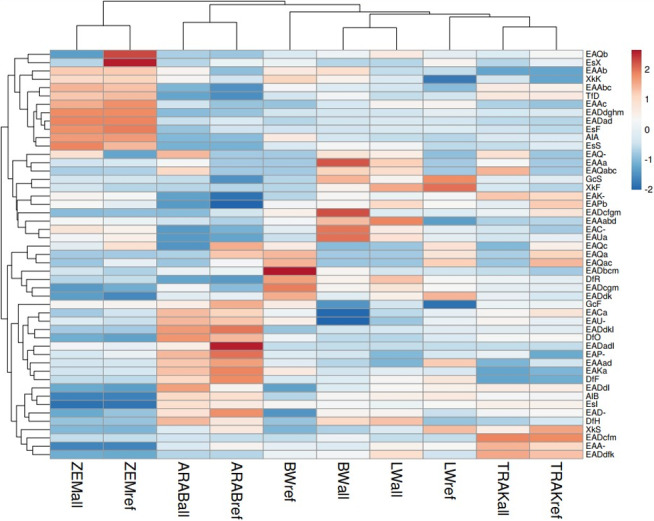
Heatmap of allele frequencies in horse populations. Rows represent alleles, and columns represent population subsets. Both were clustered using Euclidean distance and average linkage. Red indicates higher-than-average values and blue lower. “ref” – reference population (breeding horses); “all” – all analysed horses (total gene pool).

The heatmap (Fig. 4) reveals pronounced genetic shifts in the reference populations (“ref”) compared to the broader gene pools (“all”) within the same breeds. In several cases, reference groups clustered separately from the full set of analysed individuals of their respective populations, indicating recent changes in allele composition, likely driven by selection, drift, or targeted breeding strategies. This pattern is particularly evident in the BW and LW populations, suggesting divergence between historical diversity and the genetic structure of current breeding animals.

The TRAK reference and overall populations clustered closely with BW and LW subsets, reflecting their historical and ongoing contribution to the development of warmblood sport horse populations. This genetic proximity highlights shared ancestry and gene flow across these groups.

A clear separation was observed between the ZEM horses and the sport-type populations (TRAK, BW, LW), forming a distinct cluster. This confirms the unique genetic structure of this endangered native breed and is consistent with its limited gene flow from other populations. ARAB horses also formed a separate cluster, reinforcing their status as a genetically distinct, closed population.

Clustering of alleles further emphasized the variants enriched or diminished in the reference groups, pointing to alleles potentially under selection pressure or lost through genetic drift.

Overall, this heatmap visualization illustrates population restructuring under contrasting breeding systems, providing a complementary perspective to classical diversity metrics and genetic distance analyses.

## 4 Discussion

One of the fundamental aims in conservation genetics is to preserve genetic variability within populations, as it is generally assumed to be positively correlated with population viability. In the management of livestock populations, this entails minimizing the loss of genetic variation due to inbreeding and genetic drift while also preventing undesired gene flow between different breeds (Thirstrup et al., 2008; Frankham, 2010). For breeds maintained as small, closed populations, such as TRAK and ZEM, within-population diversity conservation is particularly relevant.

Our results revealed that estimates of $N_{e}$ depend heavily on the method used and the population characteristics. In TRAK, $N_{e}$ calculated via the number of reproducing animals was even lower than in the restored ZEM breed. However, $N_{e}$ estimated via the rate of inbreeding was dramatically low in ZEM, reflecting strong inbreeding pressure. This outcome is likely due to the high proportion of inbred individuals in the ZEM population and supports earlier findings that $N_{e}$ estimates are highly sensitive to pedigree completeness, mating patterns, and population structure (Leroy et al., 2013; Bokor et al., 2022). Similar patterns have been reported in endangered horse populations analysed using pedigree-based approaches, such as the Hucul and Cleveland Bay breeds, where $N_{e}$ estimates based on inbreeding rates were substantially lower and revealed stronger inbreeding pressure compared to estimates based on the number of breeding animals (Somogyvári et al., 2018; Dell et al., 2020). This is further supported by studies combining pedigree and genomic approaches, which have shown that pedigree-based estimates may underestimate inbreeding and effective population size, particularly in long-established closed populations affected by bottlenecks and hidden founder contributions (Poyato-Bonilla et al., 2022). Open populations, such as BW and LW, had high $N_{e}$ values based on breeding animal counts. However, these figures decreased significantly when estimated via ΔF , with reductions of over 60 %, highlighting unbalanced parentage and hidden relatedness. This underlines the importance of using complementary metrics, as emphasized by Martyniuk et al. (2010), to gain an accurate understanding of breed endangerment beyond FAO's basic $N_{e}$ thresholds.

The relatively high $N_{e}$ of TRAK (via breeding animals) and moderate inbreeding level ( F= 0.0373) suggest that current breeding strategies are effective in maintaining genetic diversity despite a 25 % population decline. Conservation programmes for such populations should prioritize not only the number of reproducers but also balanced parental contributions, minimization of related matings, and elongated generation intervals to slow genetic erosion (Caballero, 2020; Zechner et al., 2002). An illustrative example of the benefits of coordinated breeding is the case of the endangered Cleveland Bay, where structured mate allocation using SPARKS software increased $N_{e}$ from less than 50 to over 170 in under 2 decades (Dell et al., 2020).

The average generation interval of 12 years in TRAK supports a conservative breeding pattern, being comparable to other Lithuanian sport horse breeds and slightly higher than that of German TRAK (10 years; Teegen et al., 2009). Longer generation intervals are generally associated with slower rates of genetic change and reduced accumulation of inbreeding, which may contribute to maintaining genetic diversity in structured breeding populations (Caballero, 2020). This may help stabilize the genetic structure of the population and reduce generational drift.

The analysis of the genetic data provided additional insights into population differentiation. Similar approaches based on blood group markers have been successfully applied in endangered horse populations, such as the Hucul breed, where immunogenetic analyses revealed considerable allelic diversity and provided a basis for understanding population structure and guiding breeding strategies (Popadiuk, 2019). Importantly, the analysis included all available genotyped horses and a subset representing active breeding animals.

In TRAK, both datasets showed high allelic richness ( $N_{a}=$ 3.833 and 3.417, respectively), and observed heterozygosity increased in the reference group ( $H_{o}$ from 0.460 to 0.488). This pattern indicates successful retention – or even improvement – of genetic diversity among active reproducers, despite the constraints of a partly closed studbook. Similar trends were observed in ZEM, where $H_{o}$ and $H_{e}$ also improved compared to earlier reports (Juras and Cothran, 2005), suggesting that the implementation of structured conservation breeding is having a positive effect.

BW and LW populations exhibited the highest heterozygosity across both datasets, in line with their open breeding structure and frequent introduction of imported stallions. However, the slight decline in $A_{e}$ in reference groups hints at potential erosion of rare alleles under stabilized selection pressure. Petersen et al. (2013) similarly observed that open studbook breeds generally display higher heterozygosity but may undergo narrowing of genetic bases over time due to directional selection.

In contrast, ARAB horses showed consistently low heterozygosity and a narrow genetic base, with minimal differences between full and reference datasets. Although the total number of alleles was similar to other populations, ARAB had the highest proportion of rare alleles and lacked some variants observed in other breeds. These findings align with previous research on genetically uniform, closed populations and highlight the importance of long-term breeding coordination (Zechner et al., 2002).

Nei's genetic distances and $F_{\mathrm{st}}$ values, calculated for both datasets, revealed stable patterns of interpopulation relationships. TRAK showed the closest proximity to BW and LW, reflecting its historical influence on Lithuanian sport horse breeding. Meanwhile, the largest genetic distance was observed between TRAK and ZEM, as expected for historically and functionally distinct populations. The moderate but increasing differentiation between TRAK and ARAB ( $F_{\mathrm{st}}$ 0.015 to 0.021) in the reference dataset suggests that structured breeding in recent years is strengthening internal breed cohesion.

The assignment test further confirmed these patterns: 57.9 % of all TRAK horses were correctly assigned to their population, while this figure increased to 62.1 % in the reference group, indicating greater genetic consistency among actively bred individuals. This pattern was even more pronounced in ARAB and ZEM, where 83 % and 89 % of individuals, respectively, were assigned correctly in both datasets, consistent with their closed genetic histories and minimal admixture.

Multivariate analyses (PCA and clustering) supported the relationships described above. In the full dataset, TRAK, BW, and LW overlapped substantially, while in the reference group, population boundaries became more distinct. TRAK formed a more compact and centralized cluster, indicating increasing within-population cohesion. Heatmap analysis placed the reference populations of BW and LW as the closest relatives, with TRAK closely associated within the same cluster – consistent with historical data indicating that TRAK horses formed a significant genetic base for the Lithuanian Warmblood studbook (Šveistienė, 2007). This clustering pattern was particularly evident in the reference groups, supporting the idea that recent breeding practices have maintained historical genetic links.

The maintenance of genetic diversity within TRAK, despite its closed structure, is likely supported by the permitted inclusion of Thoroughbred and Arabian ancestry, as allowed by breeding standards (Nolte et al., 2019). This partly explains the moderate genetic proximity observed between TRAK and ARAB in our study. In contrast, native breeds such as ZEM do not benefit from this kind of gene flow, which places greater responsibility on internal diversity management.

Breeding systems and studbook structures have a profound impact on genetic diversity, influencing both within-breed variability and between-breed differentiation (Curik et al., 2017). Partly closed breeding systems, such as the TRAK studbook, are effective in maintaining breed identity but may contribute to genetic erosion over time (Leroy et al., 2013). Conversely, open systems, like those of LW and BW, enable greater genetic input from diverse sources, yet they pose challenges related to managing inbreeding and maintaining structured selection schemes (Pjontek et al., 2012). These results highlight the importance of regular genetic monitoring and tailored breeding strategies to ensure sustainable population management. The integration of genomic tools is essential for more accurate characterization of genetic architecture, controlling inbreeding and optimizing selection decisions (Yaro et al., 2017). Therefore, combining pedigree information with molecular data can support both conservation and performance goals in national horse breeding programmes.

## 5 Conclusions

Despite the limited population size, the Trakehner horse population in Lithuania demonstrated a favourable level of genetic diversity and heterozygosity. The genetic relationships observed between Trakehner and other horse populations (particularly BW and LW) are consistent with historical patterns of breed development and reflect recent gene flow. However, the observed population decline highlights the need for well-planned genetic management to prevent further erosion of genetic variation and to safeguard biodiversity.

This study underscores the importance of continued monitoring and strategic breeding efforts. While traditional selection practices have contributed to the current population structure, they may also pose risks to genetic diversity in the long term. Incorporating genomic selection tools could help to mitigate these effects by maintaining or even enhancing genetic variability. Strategic genetic management, including the use of genomic tools, should be considered an important component in efforts to preserve genetic variation and ensure the long-term sustainability of horse breeds in Lithuania.

Importantly, from 2025, national support has been introduced in Lithuania for genetic testing and parentage verification of breeding horses. This initiative is expected to facilitate the wider application of molecular tools, including microsatellite- and SNP-based analyses, in breeding programmes. The increasing availability of such data will provide a valuable foundation for more precise genetic evaluation, improved pedigree verification, and more effective long-term management of genetic diversity in Lithuanian horse populations.

## Supplement

10.5194/aab-69-323-2026-supplement The supplement related to this article is available online at https://doi.org/10.5194/aab-69-323-2026-supplement.

## Data Availability

The pedigree and genetic data used in this study are available from the corresponding author upon reasonable request.
