# Supplementary material for: Genetic variability and associations of Trakehner and other horse populations in Lithuania
Source: Arch Anim Breed. 2026 Jun 2;69(2):323–36. doi: 10.5194/aab-69-323-2026 (PMC13228154; doi:10.5194/aab-69-323-2026)
Supplement: The supplement related to this article is available online at https://doi.org/10.5194/aab-69-323-2026-supplement. [file aab-69-323-2026-supplement.pdf]

Supplement of Arch. Anim. Breed., 69, 323–336, 2026
https://doi.org/10.5194/aab-69-323-2026-supplement
© Author(s) 2026. CC BY 4.0 License.

Open Access
Archives Animal Breeding

Supplement of

Genetic variability and associations of Trakehner and other horse populations in Lithuania

Alma Račkauskaitė et al.

Correspondence to: Alma Račkauskaitė (alma.rackauskaite@lsmu.lt)

The copyright of individual parts of the supplement might differ from the article licence.

Table S1. Allelic frequencies of blood group and blood protein polymorphism systems in all and reference horses.

Locus	Allele	All horses					Signif	Reference horses					Signif
		TRAK	ARAB	BW	LW	ZEM		TRAK	ARAB	BW	LW	ZEM	
EAA	*a*	0.007	0.007	0.020	0.013	0.002	***	0.000	0.000	0.000	0.000	0.003	***$^{ARAB; TRAK; ZEM}$; ns$^{BW; LW}$
	abd	0.017	0.024	0.071	0.084	0.043		0.019	0.017	0.042	0.000	0.038	
	ad	0.318	0.459	0.342	0.319	0.357		0.358	0.483	0.375	0.455	0.359	
	b	0.012	0.038	0.046	0.023	0.051		0.012	0.017	0.042	0.023	0.051	
	bc	0.101	0.019	0.061	0.052	0.140		0.093	0.000	0.083	0.023	0.135	
	c	0.003	0.007	0.010	0.023	0.043		0.000	0.000	0.000	0.023	0.048	
	A^-	0.543	0.445	0.449	0.487	0.363		0.519	0.483	0.458	0.477	0.365	
EAC	*a*	0.464	0.490	0.403	0.439	0.434	***	0.475	0.483	0.458	0.455	0.442	***$^{ARAB; LW; TRAK; ŽEM}$; **^{BW}
	C^-	0.536	0.510	0.597	0.561	0.566		0.525	0.517	0.542	0.545	0.558	
EAD	*ad*	0.003	0.010	0.020	0.029	0.205	***$^{ARAB; LW; TRAK; ZEM}$; *^{BW}	0.000	0.033	0.000	0.023	0.205	***^{ZEM}; **^{TRAK}; ns$^{ARAB; BW; LW}$
	adl	0.000	0.012	0.000	0.000	0.008		0.000	0.033	0.000	0.000	0.006	
	bcm	0.041	0.065	0.077	0.048	0.046		0.031	0.067	0.167	0.045	0.035	
	cfgm	0.030	0.002	0.087	0.023	0.002		0.049	0.017	0.042	0.023	0.003	
	cfm	0.050	0.002	0.015	0.000	0.000		0.049	0.000	0.000	0.000	0.000	
	cgm	0.136	0.149	0.163	0.184	0.057		0.136	0.083	0.250	0.159	0.071	
	dfk	0.088	0.017	0.046	0.068	0.001		0.080	0.017	0.042	0.023	0.000	
	dghm	0.096	0.005	0.092	0.084	0.388		0.111	0.017	0.083	0.136	0.391	
	dk	0.134	0.125	0.128	0.158	0.066		0.117	0.133	0.208	0.205	0.054	
	dkl	0.043	0.151	0.026	0.032	0.004		0.043	0.167	0.000	0.045	0.006	
	dl	0.144	0.200	0.102	0.129	0.046		0.142	0.133	0.042	0.136	0.038	
	D^-	0.235	0.262	0.245	0.245	0.176		0.241	0.300	0.167	0.205	0.189	
EAK	*a*	0.002	0.055	0.020	0.026	0.019	ns	0.006	0.067	0.042	0.023	0.022	ns
	K^-	0.998	0.945	0.980	0.974	0.981		0.994	0.933	0.958	0.977	0.978	
EAP	*b*	0.038	0.012	0.046	0.058	0.045	ns	0.062	0.000	0.042	0.045	0.038	ns
	P^-	0.962	0.988	0.954	0.942	0.955		0.938	1.000	0.958	0.955	0.962	
EAQ	*a*	0.002	0.007	0.000	0.000	0.001	***	0.111	0.117	0.125	0.091	0.000	***$^{LW; TRAK; ZEM}$; **^{ARAB}; ns^{BW}
	abc	0.083	0.067	0.071	0.065	0.001		0.000	0.000	0.000	0.000	0.003	
	ac	0.000	0.002	0.000	0.000	0.003		0.204	0.100	0.208	0.182	0.077	
	b	0.172	0.127	0.199	0.239	0.077		0.210	0.117	0.167	0.182	0.372	
	c	0.184	0.101	0.189	0.219	0.357		0.475	0.667	0.500	0.545	0.548	
	Q^-	0.560	0.695	0.541	0.477	0.561		0.111	0.117	0.125	0.091	0.000	
EAU	*a*	0.089	0.024	0.250	0.171	0.129	***^{BW}; **^{ZEM}; *^{LW}; ns$^{ARAB; TRAK}$	0.117	0.033	0.083	0.114	0.151	*^{ZEM}; ns$^{ARAB; BW; LW; TRAK}$
	U^-	0.911	0.976	0.750	0.829	0.871		0.883	0.967	0.917	0.886	0.849	
Al	*A*	0.535	0.421	0.520	0.494	0.821	***	0.543	0.433	0.667	0.477	0.821	***$^{ARAB; LW; TRAK}$; *^{ZEM}; ns^{BW}
	B	0.465	0.579	0.480	0.506	0.179		0.457	0.567	0.333	0.523	0.179	
Gc	*F*	0.887	0.913	0.796	0.855	0.902	**^{TRAK} ns$^{ARAB; BW; LW; ZEM}$	0.889	0.967	0.917	0.773	0.905	*^{TRAK} ns$^{ARAB; BW; LW; ZEM}$
	S	0.113	0.087	0.204	0.145	0.098		0.111	0.033	0.083	0.227	0.095	

Es	*F*	0.083	0.038	0.082	0.106	0.334	***$^{ZEM;}$	0.068	0.083	0.083	0.114	0.349	*$^{LW;}$
	I	0.892	0.962	0.852	0.832	0.523	ns $^{ARAB; BW; LW;}$	0.889	0.917	0.875	0.864	0.532	ns $^{ARAB; BW;}$
	S	0.025	0.000	0.066	0.061	0.141	^{TRAK}	0.043	0.000	0.042	0.023	0.119	$^{TRAK; ŽEM}$
	X	0.000	0.000	0.000	0.000	0.002		0.068	0.083	0.083	0.114	0.349	
Xk	*F*	0.003	0.000	0.010	0.019	0.002	ns	0.000	0.000	0.000	0.023	0.000	ns
	K	0.952	0.976	0.980	0.958	0.997		0.926	0.950	1.000	0.909	1.000	
	S	0.045	0.024	0.010	0.023	0.001		0.074	0.050	0.000	0.068	0.000	
Tf	*D*	0.369	0.084	0.296	0.190	0.451	***^{ARAB}	0.364	0.050	0.208	0.205	0.462	ns
	F	0.326	0.558	0.429	0.487	0.393	**$^{LW; TRAK}$	0.333	0.633	0.417	0.523	0.391	
	H	0.002	0.024	0.020	0.023	0.000	*^{ZEM}	0.006	0.017	0.000	0.000	0.000	
	O	0.219	0.327	0.153	0.152	0.114	ns^{BW}	0.222	0.300	0.208	0.182	0.103	
	R	0.084	0.007	0.102	0.148	0.041		0.074	0.000	0.167	0.091	0.045	

TRAK = Trakehner; ARAB = Arabian; BW = Baltic Warmblood; LW = Lithuanian Warmblood; ZEM = Zemaitukai; Signif = significance for HWE; ns = not significant; * $P < 0.05$; ** $P < 0.01$; *** $P < 0.001$.
